# Behavioral and Neurochemical Effects of Alpha-Lipoic Acid in the Model of Parkinson's Disease Induced by Unilateral Stereotaxic Injection of 6-Ohda in Rat

**DOI:** 10.1155/2013/571378

**Published:** 2013-08-19

**Authors:** Dayane Pessoa de Araújo, Caren Nádia Soares De Sousa, Paulo Victor Pontes Araújo, Carlos Eduardo de Souza Menezes, Francisca Taciana Sousa Rodrigues, Sarah Souza Escudeiro, Nicole Brito Cortez Lima, Manoel Claúdio Azevedo Patrocínio, Lissiana Magna Vasconcelos Aguiar, Glauce Socorro de Barros Viana, Silvânia Maria Mendes Vasconcelos

**Affiliations:** ^1^Department of Physiology and Pharmacology, Faculty of Medicine, Federal University of Ceará, Rua Cel. Nunes de Melo 1127, 60431-270 Fortaleza, CE, Brazil; ^2^Christus Faculty of Medicine, Avenida Dom Luiz 911, 60160-230 Fortaleza, CE, Brazil

## Abstract

This study aimed to investigate behavioral and neurochemical effects of **α**-lipoic acid (100 mg/kg or 200 mg/kg) alone or associated with L-DOPA using an animal model of Parkinson's disease induced by stereotaxic injection of 6-hydroxydopamine (6-OHDA) in rat striatum. Motor behavior was assessed by monitoring body rotations induced by apomorphine, open field test and cylinder test. Oxidative stress was accessed by determination of lipid peroxidation using the TBARS method, concentration of nitrite and evaluation of catalase activity. **α**-Lipoic acid decreased body rotations induced by apomorphine, as well as caused an improvement in motor performance by increasing locomotor activity in the open field test and use of contralateral paw (in the opposite side of the lesion produced by 6-OHDA) at cylinder test. **α**-lipoic acid showed antioxidant effects, decreasing lipid peroxidation and nitrite levels and interacting with antioxidant system by decreasing of endogenous catalase activity. Therefore, **α**-lipoic acid prevented the damage induced by 6-OHDA or by chronic use of L-DOPA in dopaminergic neurons, suggesting that **α**-lipoic could be a new therapeutic target for Parkinson's disease prevention and treatment.

## 1. Introduction

Parkinson's disease (PD) is defined as a progressive neurological disorder characterized by degeneration of dopaminergic neurons in the substantia nigra pars compacta and *locus coeruleus*. This degeneration results in decreased production of dopamine (DA), producing a cluster of symptoms characterized mainly by motor disturbances [1, 2].

The current treatment is limited only to relief of symptoms and to delay the velocity of neurodegeneration [3]. L-DOPA is currently the most effective drug against the motor symptoms of PD. It is precursor of DA, metabolized to 3-O-methyldopa (3-OMD) and rapidly decarboxylated to DA in brain [4].

Initial treatment with L-DOPA causes a significant improvement of PD symptoms, however, with progression of disease, L-DOPA loses its efficiency and effectiveness, being necessary to increase the dose, replace or combine a therapy [5].

Chronic use of L-DOPA, is associated by the development of adverse events related to fluctuations in motor response. These motor fluctuations include on-off fluctuations (ON—the period when the drug is effective; OFF—when it is ineffective), sudden, unpredictable changes in mobility, and the wearing-off phenomenon, a decrease in the duration of action of levodopa. But, the most dramatic class of motor fluctuation is involuntary movements known as l-dopa-induced dyskinesia [4, 6, 7].

These adverse events limit the use of L-DOPA and makes impossible to continue the treatment. Therefore, neuroprotective strategies are being proposed as molecular mechanisms involved in PD pathogenesis are being elucidated, among which we quote the *α*-lipoic acid (LA) as a new therapeutic approach complementary to the current treatment of PD [8].

LA is an antioxidant naturally synthesized in human body. In its structural formula, there are two thiol groups that can be oxidized or reduced thus, it is a redox couple. Both the oxidized form LA and the reduced form dihydrolipoic acid (DHLA) act as antioxidant [9].

LA is an amphipathic substance, soluble in water and fat, and therefore can neutralize free radicals in aqueous or lipidic regions of cells [10]. Furthermore, DHLA is able to regenerate other antioxidants with low molecular weight, such as glutathione (GSH), coenzyme Q10 and vitamins A and C [11]. It is also attributed to this substance an anti-inflammatory activity, and therefore the effect of short- and long-term reduction in oxidative processes related to neurodegenerative diseases. Moreover, it works as a metal chelator, reducing reactive oxygen species (ROS) production [9].

Therefore, the aim of this study was to investigate neurochemical and behavioral effects of LA alone or in combination with L-DOPA using an animal model of PD induced by the stereotaxic injection of the neurotoxin 6-hydroxydopamine(6-OHDA) in rat striatum.

## 2. Materials and Methods

### 2.1. Animals

Male Wistar rats (weighing 250–300 g), 8 per group, were used for behavioral and neurochemical tests. The animals were housed in standard environmental conditions (22 ± 1°C, humidity 60 ± 5%, 12 h light: 12 h dark cycle) with free access to water and food. All experiments were performed in accordance with the NIH Guide for Care and Use of Laboratory Animals.

### 2.2. Drugs

*α*-Lipoic acid, 6-hydroxydopamine and ascorbic acid were purchased from Sigma (USA). Apomorphine were purchased from Tocris (USA). L-3,4-Dihydrophenylalanine (L-DOPA) and carbidopa (Carbidol 250/25 mg) were purchased from Teuto (Brazil).

### 2.3. Experimental Protocol

The animals were divided into seven groups and received saline and 5% cellulose (Control and 6-OHDA group), LA (100 or 200 mg/Kg), or L-DOPA (50 mg/Kg) by the oral route (p.o.). In groups of association, the animals were pretreated with LA (100 or 200 mg/Kg, p.o.) one hour before administration of L-DOPA (50 mg/Kg). After one hour of the last treatment, the animals were anesthetized with a combination of ketamine (100 mg/kg, intraperitoneally, i.p.) and xylazine (5 mg/kg, i.p.) and given a unilateral injection of 1 *μ*L of 6-OHDA (12 *μ*g/*μ*L per site) with 0.2 mg/mL L-ascorbic acid (Sigma Chemical Co., St. Louis, MO, USA), into the right striatum. Unilateral, intrastriatal 6-OHDA injection was performed through a 10 *μ*L Hamilton syringe using a stereotaxic apparatus (Stoelting, USA) at the following coordinates (mm): site 1: L: −2.5, AP: +0.5, V: +5.0; site 2: L: −3.0, AP: −0.5, V: +6.0; and site 3: L: −3.7, AP: −0.9, V: +6.5 from the bregma, according to the Atlas of Paxinos and Watson (1986).

The treatment with LA (100 or 200 mg/Kg) or L-DOPA (50 mg/Kg) or with the association of LA (100 or 200 mg/Kg) with L-DOPA (50 mg/Kg) continued daily for 14 days. Twenty-four hours after the last drug administration (2 weeks after the 6-OHDA injection) the animals were subjected to behavioral tests.

### 2.4. Analysis of Motor Behavior

#### 2.4.1. Open-Field Test (OF)

The OF area was made of acrylic (transparent walls and black floor, 50 cm × 50 cm × 50 cm) divided into four squares of equal area. The OF was used to evaluate the exploratory activity of the rats. Each rat was placed in the center of the arena and the number of squares crossed, with the four paws (locomotor activity) was recorded for 5 min after a minute of habituation. Before introducing each animal, the arena was cleaned with 5% alcohol to eliminate the possible bias due to the odor that could be left by previous animals [12].

#### 2.4.2. Cylinder Test

The cylinder test aims to evaluate the asymmetry in forelimb use in vertical exploratory activity (rearing). The animals were placed into a cylinder of 20 cm diameter and 40 cm high. Each animal was individually evaluated for 5 minutes. The number of contacts on the cylinder wall with the right paw, left and both paws simultaneously were counted. The results were given in percentage and calculating was done as follows: the total sum of contacts on the cylinder wall with the right paw, left paw and both totaled 100%, based on this, it is calculated the percentage value for each finding [13].

#### 2.4.3. Rotational Behavior

The behavior was assessed by monitoring body rotations induced by apomorphine (3 mg/kg, i.p.). The number of net rotations (the number of 360° contralateral turns) was recorded for 60 min and, at the next day, animals were sacrificed, and the striatal tissue was collected and stored at −70°C until use.

### 2.5. Neurochemical Study

#### 2.5.1. Evaluation of Lipid Peroxidation

Brain areas, the prefrontal cortex (PFC), hippocampus (HC) and striatum (s) from all groups were dissected to prepare 10% homogenates (w/v, in 1.15% KCl). The formation of lipid peroxides during lipid peroxidation was followed by measuring the thiobarbituric acid reactive substances (TBARS), as previously described by Draper and Hadley [14]. Briefly, samples were mixed with 1 mL of 10% trichloroacetic acid and 1 mL of 0.6% thiobarbituric acid. The reaction media was heated in a boiling water bath for 15 min, and n-butanol (2 : 1 v/v) was added to the media. After centrifugation (800 ×g, 5 min), TBARS contents were determined at 535 nm.

#### 2.5.2. Nitrite Determination

Tissue samples from PFC, HC or S were used to prepare 10% homogenates (w/v). After centrifugation (800 ×g, 10 min), supernatants were collected and the NO production was determined by the Griess reaction [15]. Briefly, 100 *μ*L of the supernatant were incubated with 100 *μ*L of the Griess reagent [1% sulfanilamide in 1% H_3_PO_4_/0.1% N-(1-naphthyl)-ethylenediamine dihydrochloride/1% H_3_PO_4_/distilled water (1 : 1 : 1 : 1)] at room temperature for 10 min. The absorbance was measured at 550 nm via microplate reader. Nitrite concentration (*μ*M) was determined from a standard NaNO_2_ curve.

#### 2.5.3. Evaluation of Catalase Activity

Catalase activity was measured by the method that employs hydrogen peroxide to generate H_2_O and O_2_ [16]. The activity was measured by the degree of this reaction. The standard assay substrate mixture contained 0.30 mL of hydrogen peroxide in 50 mL of 0.05 M phosphate buffer, pH 7.0. The sample aliquot (20 *µ*L) was added to 980 *µ*L of the substrate mixture. After 1 min, the initial absorbance was recorded and the final absorbance was read after 6 min. The reaction was followed at 230 nm. A standard curve was established using purified catalase (Sigma, MO, USA) under identical conditions. All samples were diluted in 0.1 mmol/L phosphate buffer (pH 7.0) to provoke an inhibition of 50% of diluent rate (i.e., the uninhibited reaction) and results were expressed as *µ*M/min/*µ*g protein.

### 2.6. Statistical Analysis

All tests were analyzed by One-way ANOVA using Prism 5.0 software. For meaningful results, multiple comparisons were made by the Tukey as the *post hoc* tests. Results were considered significant at *P* < 0.05 and presented as mean ± SEM.

## 3. Results

### 3.1. Behavioral Tests

#### 3.1.1. Rotational Behavior

**Figure 1:**
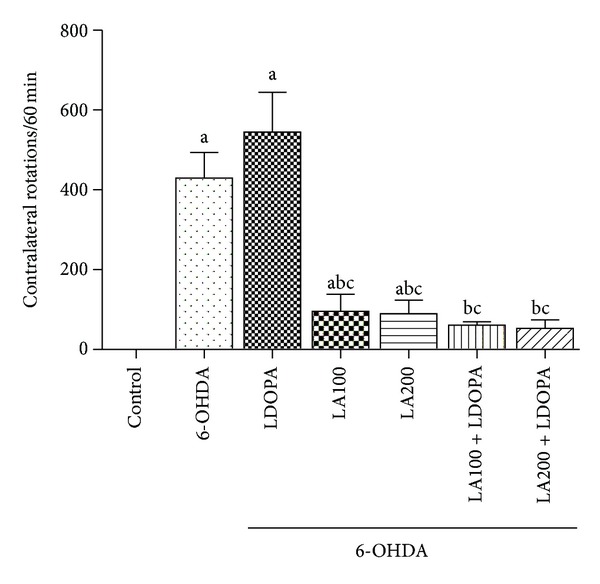
Determination of the rotational behavior induced by apomorphine (1 mg/kg i.p.) 60 min in rats subjected to the pretreatment with 6-OHDA treated with *α*-lipoic acid at doses of 100 or 200 mg/Kg alone or in combination with L-DOPA (50 mg/Kg). Eight animals per group were used. Values are expressed as mean ± S.E.M of the number of experiment. a *versus* control, b *versus* 6-OHDA, and c *versus * 6-OHDA + L-DOPA respectively; at *P* < 0.0001 (one-way ANOVA and Tukey as the *post hoc* tests).

The results showed a drastic increase in body rotation, induced by the 6-OHDA lesion, after the apomorphine challenge (positive control) as compared to the control group (Control: 0.00 ± 0.00, 6-OHDA: 429.5 ± 26.27 turns/h). The number of rotations per hour decreased by 70% and 80% in the 6-OHDA-lesioned group, after the treatment with LA100 (95.67 ± 17.51 turns/h) and LA200 (89.33 ± 14.15 turns/h). Our results showed that LA significantly diminishes the apomorphine-induced rotations resulted from the striatal 6-OHDA lesion, and partially reversed the neurotoxin effects. However, the LA effect on body rotation in the 6-OHDA group was not altered by its association with L-DOPA (6-OHDA + L-DOPA: 545.2  ±  44.32 turns/h) (Figure 1).

In the striatal 6-OHDA-lesioned group treated with association of *α*-lipoic acid and L-DOPA (6-OHDA + LA100 + L-DOPA: 61.17 ± 3.54; 6-OHDA + LA200 + L-DOPA: 53.33  ±  8.45 turns/h) LA was able to reverse the increase in the number of contralateral rotations induced by apomorphine when compared to the 6-OHDA (429.5 ± 26.27 rotations/h) and the group of L-DOPA alone (6-OHDA + L-DOPA: 545.2 ± 44.32 rotations/h) [*F*(6,40) = 109.8, *P* < 0.0001].

#### 3.1.2. Open Field Test

**Figure 2:**
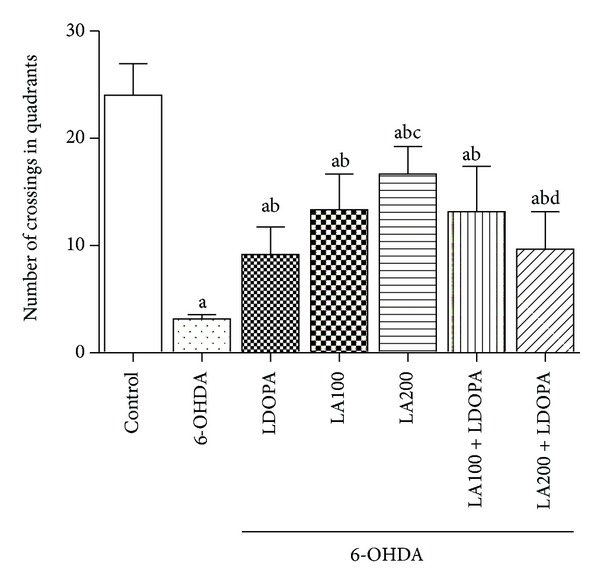
Determination of the effect of *α*-lipoic acid alone or in combination with L-DOPA in the number of crossings in the open field test in animals pretreated with 6-OHDA. Eight animals per group were used. Values are expressed as mean ± S.E.M of the number of experiments. a *versus* control, b *versus* 6-OHDA, c *versus* 6-OHDA + LDOPA, d *versus* 6-OHDA + LA200, respectively; at *P* < 0.0001 (one-way ANOVA followed by Tukey's *post hoc* test).

The results indicated significant differences in the locomotor parameter in the open field test. The 6-OHDA-lesioned group (3.16 ± 0.16) showed a decline in the locomotor activity when compared to the control group (24 ± 1.21). The groups treated with LA (6-OHDA + LA100: 13.33 ± 1.35; 6-OHDA + LA200: 16.67 ± 1.05) showed an increase in the number of crossing when compared to the 6-OHDA. The associations of *α*-lipoic acid with L-DOPA (6-OHDA + LA100 + L-DOPA: 13.13 ± 1.72; 6-OHDA + LA200 + L-DOPA: 9.66 ± 1.43) also showed an increase in the locomotor activity as compared to the group of 6-OHDA (Figure 2).

However, the group treated with *α*-lipoic acid at the dose of 200 mg/Kg associated to L-DOPA (6-OHDA + LA200 + L-DOPA: 9.66 ± 1.43) showed a decrease (40%) in the locomotor activity as compared to the group treated with *α*-lipoic acid alone at the dose of 200 mg/Kg (6-OHDA + LA200: 16.67 ± 1.05) [*F*(6.41) = 28.38; *P* < 0.0001] (Figure 2).

**Figure 3:**
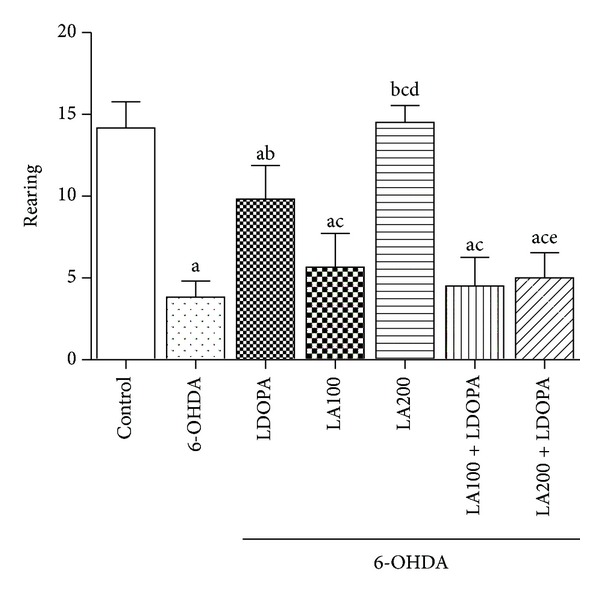
Determination of the effect of *α*-lipoic acid alone or associated with L-DOPA on the number of rearing in the open field test in animals pretreated with 6-OHDA. Values are expressed as mean ± SEM of the number of observations. Eight animals per group were used. a *versus* control, b *versus* 6-OHDA, c *versus* 6-OHDA + LDOPA, d *versus* 6-OHDA + LA100, e *versus *6-OHDA + LA200 respectively; at *P* < 0.0001 (one-way ANOVA followed by Tukey's *post hoc* test).

In the assessment of vertical exploratory activity (rearing) the results showed that the 6-OHDA-lesioned group (3.83 ± 0.4) without further treatment showed a decrease (72%) in the frequency of rearing when compared to the control group (14.17 ± 0.65). In the 6-OHDA-lesioned groups treated with either LA or L-DOPA or associations of LA with L-DOPA, only the group of LA200 (6-OHDA + LA200: 14.5 ± 0.42) and L-DOPA (6-OHDA + L-DOPA: 9.83 ± 0.83) showed an increase in the number of rearing as compared to 6-OHDA lesioned group (Figure 3).

The group treated with LA200 (6-OHDA + LA200: 14.5 ± 0.42) increased the number of rearing as compared to L-DOPA group (6-OHDA + L-DOPA: 9.83 ± 0.83). However, groups treated with associations LA and L-DOPA (6-OHDA + LA100 + L-DOPA: 4.5 ± 0.71; 6-OHDA + LA200 + L-DOPA: 5 ± 0.63) decreased the rearing when compared to L-DOPA group (6-OHDA + L-DOPA: 9.83 ± 0.83) [*F*(6.41) = 48.02, *P* < 0.0001].

#### 3.1.3. Cylinder Test

The cylinder test is used as a tool for assessing fine motor activity, through the percentage of times the animal presents vertical exploratory activity (rearing), touches the cylinder wall with the front legs [ipsilateral lesion (IP), contralateral lesion (CP) and with both legs simultaneously (2P)]. In the control group most animals touched the cylinder wall with both paws simultaneously (Control IP: 10.2%, CP: 7.5%; 2P: 82.3%). Regarding the group of 6-OHDA all animals touched the cylinder wall with IP paws (6-OHDA IP: 100%, CP: 0.0% 2P: 0.0%) (Table 1).

The percentage of touches with both paws simultaneously in the cylinder wall increased in animals treated with the *α*-lipoic acid (6-OHDA + LA100: 21.9%; 6-OHDA + LA200: 15%) and in association (6-OHDA + LA100 + L-DOPA: 52.8%) as compared with 6-OHDA lesioned (0.0%) or L-DOPA group (6-OHDA + L-DOPA: 4%) [*F*(6.41) = 181.7, *P* < 0.0001] (Table 1).

Animals treated with *α*-lipoic acid (6-OHDA + LA100: 16.2%; 6-OHDA + LA200: 18.3%) and the associations (6-OHDA + LA100 + L-DOPA: 34.2%; 6-OHDA + LA200 + L-DOPA: 17.2%) showed a significant improvement in the fine motor activity since the animals touches the cylinder wall with the paw contralateral to the lesion compared to the group of 6-OHDA (0.0%) or L-DOPA (6-OHDA + L-DOPA: 4.59%) (Table 1).

The group treated with association LA100 + L-DOPA (6-OHDA + LA100 + L-DOPA: 31.3%) increased the use of the paw contralateral the lesion in relation to the group LA100 or LA200 alone and association LA200 + L-DOPA ([*F*(6.41) = 15.98, < 0.0001]).

### 3.2. Neurochemical Study

#### 3.2.1. Concentration of Lipid Peroxidation (TBARS)

**Figure 4:**
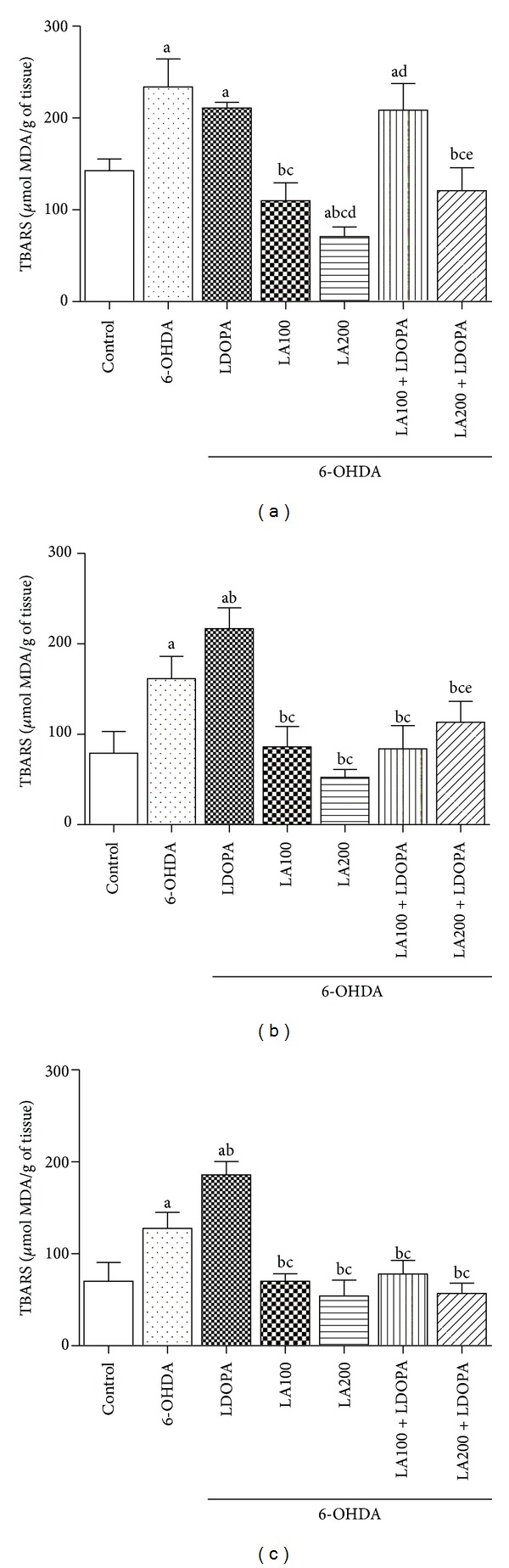
Effect of *α*-lipoic acid alone or in combination with L-DOPA on lipid peroxidation in the prefrontal cortex (a), hippocampus (b) and striatum (c) of rats pre-treated with 6-OHDA. Eight animals per group were used. Values are expressed as mean ± S.E.M of the TBARS quantities expressed in MDA/g tissue. a *versus* control, b *versus* 6-OHDA, c *versus* 6-OHDA + LDOPA, d *versus* 6-OHDA + LA100, e *vesus *6-OHDA + LA200 respectively; at *P* < 0.0001 (one-way ANOVA followed by Tukey's *post hoc* test).

Figure 4 show the effects of chronic administration of *α*-lipoic acid (100 or 200 mg/kg) alone or in combination with L-DOPA (50 mg/Kg) on the malondialdehyde (MDA) content. The results showed that exposure of cells of the PFC, HC and S to 6-OHDA (234 ± 12.46; 161.6 ± 10.01; 127.6 ± 7.15, resp.) or L-DOPA (6-OHDA + L-DOPA: 210.8 ± 2.60; 216.9 ± 9.41; 186  ±  5.87, resp.) caused an increase in the MDA content when compared with the control group (142.6 ± 5.17; 79.05 ± 9.79; 70.19 ± 8.38, resp.) demonstrating that oxidative stress plays an important role in the mechanism of lesion induced by 6-OHDA.

The CPF of the groups treated with LA100 (6-OHDA + LA100: 109.8 ± 7.94) or LA200 (6-OHDA + LA200: 70.92 ± 4.29) showed a significant reduction in the MDA levels compared to the 6-OHDA group (234 ± 12.46) or L-DOPA (6-OHDA + L-DOPA: 210.8 ± 2.60). In groups treated with the association was observed reduction of [MDA] only at the dose of 200 (6-OHDA + LA200 + L-DOPA: 120.9 ± 10.18) as compared to 6-OHDA e L-DOPA (Figure 4(a)).

The groups treated with LA (6-OHDA + LA100: 109.8 ± 7.94; 6-OHDA + LA200: 70.92 ± 4.29) presented better responses in decreasing oxidative stress when compared to the groups of associations (6-OHDA + LA100 + L-DOPA: 208.6 ± 11.87; 6-OHDA + LA200 + L-DOPA: 120.9 ± 10.18) in the PFC (Figure 4(a)).

The concentrations of MDA in HC and S were highest in the L-DOPA group (6-OHDA + L-DOPA: 216.9 ± 9.41; 186 ± 5.87, resp.) compared with 6-OHDA group (6-OHDA: 161.6 ± 10.01; 127.6 ± 7.15, resp.).

The treatment of 6-OHDA-lesioned rats with LA100 or 200 mg/Kg reduced lipid peroxidation in the HC (6-OHDA + LA100: 86.06  ±  9.19; 6-OHDA + LA200: 52.38 ± 3.54) and S (6-OHDA + LA100: 70.18 ± 3.33; 6-OHDA + LA200: 54.15 ± 7.06) compared to the 6-OHDA-lesioned group (HC:161.6 ± 10.01;   S:127.6 ± 7.15) or L-DOPA (6-OHDA+L-DOPA: 216.9 ± 9.41; 186 ± 5.87, resp.) (Figures 4(b) and 4(c)).

Similar effects were observed with associations in the HC (6-OHDA + LA100 + L-DOPA: 83.99 ± 10.44; 6-OHDA + LA200 + L-DOPA: 113.4 ± 9.34) or S (6-OHDA + LA100 + L-DOPA: 78.20 ± 5.95; 6-OHDA+LA200+ L-DOPA: 56.82 ± 4.59), as compared to 6-OHDA or L-DOPA group [HC: *F*(6.41) = 39.42, *P* < 0.0001] or [S: *F*(6.41) = 59.31, *P* < 0.0001] (Figures 4(b) and 4(c)).

#### 3.2.2. Nitrite Determination

**Figure 5:**
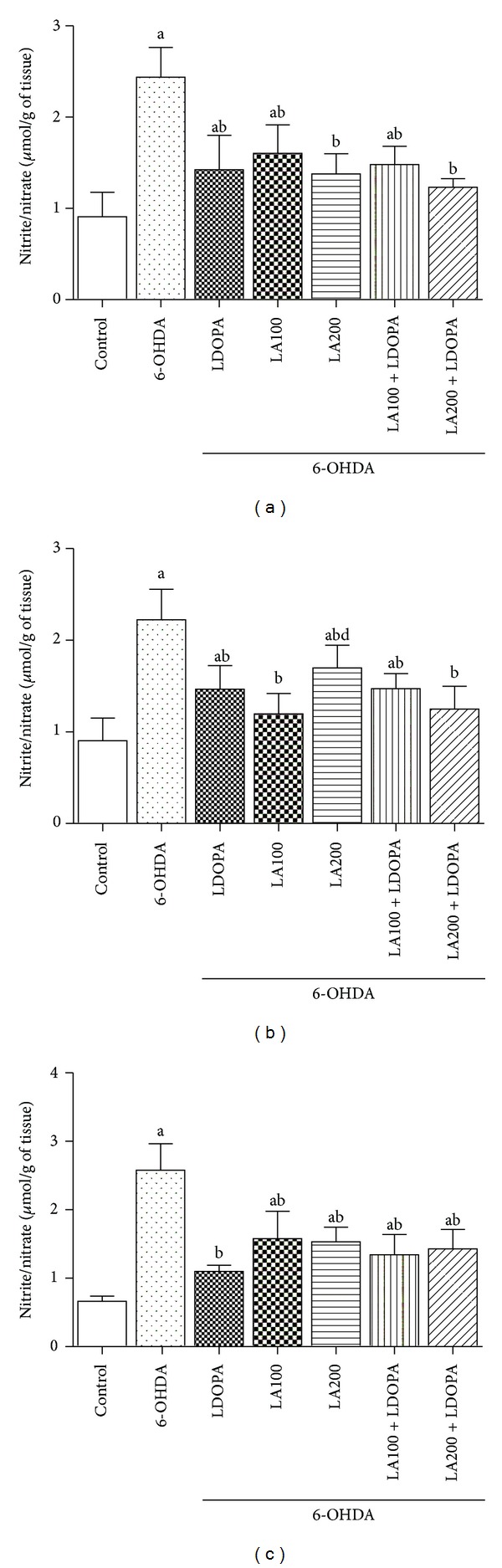
Effect of *α*-lipoic acid alone or in combination with L-dopa of the concentration of nitrite/nitrate in the prefrontal cortex (a), hippocampus (b) and striatum (c) of rats pre-treated with 6-OHDA. Eight animals per group were used. Values represent mean ± SEM of the quantities of nitrite/nitrate expressed in *µ*mol/g of tissue. a *versus* control, b *versus* 6-OHDA respectively; at *P* < 0.0001 (one-way ANOVA followed by Tukey's *post hoc* test).

The results showed that concentrations of nitrite/nitrate in the 6-OHDA group increased in the PFC (2.44 ± 0.12), HC (2.31 ± 0.13) and S (2.55 ± 0.11) when compared to control group (0.90 ± 0.1, 0.90 ± 0.1, 0.64 ± 0.02, resp.) (Figure 5).

Treatment of 6-OHDA-lesioned rats with LA caused a reduction of nitrite/nitrate levels [PFC (6-OHDA + LA100: 1.6 ± 0.12; 6-OHDA + LA200: 1.38 ± 0.08), HC (6-OHDA + LA100: 1.19 ± 0.09; 6-OHDA + LA200: 1.69 ± 0.1), S (6-OHDA + LA100: 1.57 ± 0.16; 6-OHDA + LA200: 1.53 ± 0.08)], it was also observed in association with L-DOPA [PFC (6-OHDA + LA100 + L-DOPA: 1.48 ± 0.08; 6-OHDA + LA200 + L-DOPA: 1.23 ± 0.03), HC (6-OHDA + LA100 + L-DOPA: 1.47 ± 0.06; 6-OHDA + LA200 + L-DOPA: 1.24 ± 0.1), S (6-OHDA + LA100 + L-DOPA: 1.34 ± 0.12; 6-OHDA + LA200 + L-DOPA: 1.42 ± 0.11)] compared with 6-OHDA-lesioned group (Figure 5).

#### 3.2.3. Evaluation of Catalase Activity

**Figure 6:**
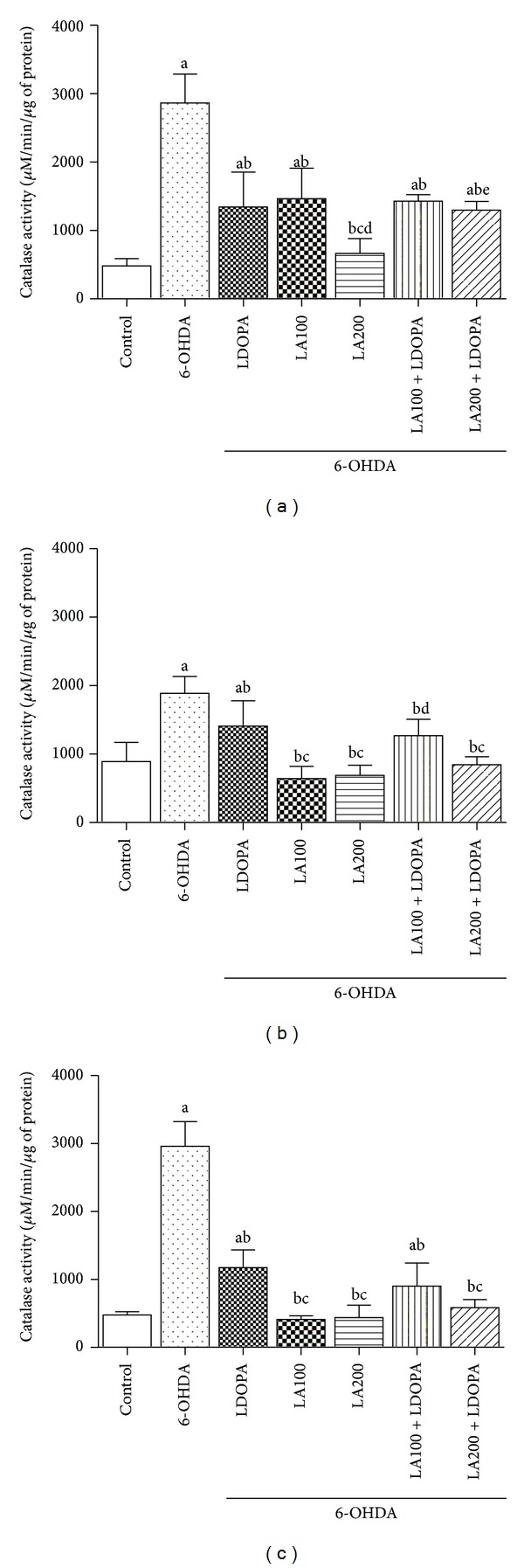
Effects of *α*-lipoic acid alone or in combination with L-DOPA on the activity of catalase in the prefrontal cortex, hippocampus and striatum of rats subjected to pre-treatment with 6-OHDA. Eight animals per group were used. Values represent mean ± SEM of enzyme activity expressed in *µ*M/min/*µ*g of tissue. a *versus* control, b *versus* 6-OHDA, c *versus* 6-OHDA + LDOPA, d *versus* 6-OHDA + LA100, e *versus *6-OHDA + LA200 respectively; at *P* < 0.0001 (one-way ANOVA followed by Tukey's *post hoc* test).

Increases in catalase activity in the PFC, HC and S (Figure 6) was observed in 6-OHDA or L-DOPA group when compared with control (PFC: 480.8 ± 43.89, HC: 890.3 ± 114.5; S: 478.1 ± 19.33).

On the other hand, with the 6-OHDA group treated with LA100 (HC: 641.7 ± 73.29; S: 410.4 ± 22.62) or LA200 (HC: 686.8 ± 61.38; S: 436.9 ± 75.09) decreases in catalase activity compared with 6-OHDA in the HC and S (HC: 1890 ± 100.4; S: 2961 ± 148.4; PFC: 2865 ± 173.2) were observed. But in PCF this effect occurred only with the group LA200 (PCF: 663.9 ± 89.38).

Similarly, decreases in the catalase activity were observed with associations LA100 + L-DOPA [PFC (6-OHDA + LA100 + L-DOPA: 1429 ± 38.8); HC (6-OHDA + LA100 + L-DOPA: 1268  ±  98.49); S (6-OHDA + LA100 + L-DOPA: 903.1 ± 138.4)] or LA200 + L-DOPA [PFC (6-OHDA + LA200 + L-DOPA: 1297 ± 51.89); HC (6-OHDA + LA200 + L-DOPA: 844.6 ± 48); S(6-OHDA + LA200 + L-DOPA: 585.5 ± 48.41)] when compared to the group of 6-OHDA (PFC: 2865 ± 173.2; HC: 1890 ± 100.4; S: 2961 ± 148.4) (Figure 6).

In HC [*F*(6.41) = 21.53, < 0.0001] and S [*F*(6.41) = 94.93, *P* < 0.0001], the association with higher dose (6-OHDA + LA200 + L-DOPA) reduced catalase activity when compared with the group of L-DOPA alone, showing that LA probably reduced the need for activation of this enzyme by reducing oxidative stress (Figures 6(b) and 6(c)).

## 4. Discussion

Antioxidants have been used as a tool for combating neurodegenerative diseases, since oxidative stress is one of the mechanisms involved in the pathogenesis of various central nervous systems (CNS) disorders. *α*-Lipoic acid, as an antioxidant, has been investigated as a new therapeutic alternative for diseases such as Alzheimer's disease, depression, ischemia and PD among others [17–22].

Parkinson's disease (PD) is a neurodegenerative disorder that is pathologically characterized by widespread, progressive neural degeneration and neuronal death, affecting dopamine-producing neurons whose cell bodies locate to within the compacta part of the substantia nigra (SNpc), and also other dopamine-rich areas, such as the olfactory tubercles and frontal cortex [23].

One of the most frequently used toxins in rodent models of PD is the neurotoxin 6-OHDA [24]. 6-OHDA is a neurotoxin that selectively destroys catecholaminergic neurons and it is typically injected unilaterally, since bilateral injections cause high mortality. This model provides easier evaluation of physical disabilities, by using assays which examine a bias side, for example, rotation tests drug-induced and spontaneous motor tests [25]. Furthermore, intraestriatal injection of 6-OHDA has been shown to permanently degenerate practically all dopaminergic neurons in the SN pars compacta leading to stable motor deficits over time [26].

 Previous studies have shown that 6-OHDA is transported into dopaminergic neurons where it is oxidized to produce hydrogen peroxide, superoxide, and hydroxyl radicals [27–29]. 6-OHDA injection results in the formation of reactive oxygen species (ROS), and produces a potent inhibition of the mitochondrial respiratory chain complexes I and IV, *in vitro *[30].

The present study examined the effects of *α*-lipoic acid in the model of Parkinson's disease induced by the unilateral striatal injection (right hemisphere) with 6-OHDA. The results showed that the 6-OHDA produced an increase in the number of rotations (contralateral to the lesion) induced by apomorphine. This is probably caused by sensitization and increase in the number of dopamine receptors on the side opposite to the lesion, due to the loss of dopaminergic terminals in the striatum lesioned with 6-OHDA [31].

Lesions of the nigrostriatal system, resulting from auto-oxidation of 6-OHDA, are directly related to the increase in dopaminergic stimulation via adenylyl cyclase in D1 receptors [31]. The animal model of PD, induced by this neurotoxin, shows behavioral changes with treatment with D1 receptor agonists resulting in up-regulation of these receptors as a compensatory mechanism for the loss of dopaminergic neurons caused by 6-OHDA [32].

Our results demonstrated that *α*-lipoic acid partially reversed the impairment produced by 6-OHDA by promoting a significant reduction in rotational behavior, as well as increasing the locomotor activity in the open field test. This effect may be related to the antioxidant activity of *α*-lipoic acid that possibly decreased the process of sensitization of dopaminergic receptors resulting in decreased apomorphine-induced rotations [33].

Recently, Jalali-Nadoushan and Roghani [34] demonstrated that *α*-lipoic acid pretreatment significantly attenuated rotations and prevented loss of SNC neurons. They suggested that the *α*-lipoic acid may confer neuroprotection against the neurotoxicity of 6-OHDA and this is due in part by attenuation of oxidative stress burden.

In a study by Zaitone et al. [35] in an animal model of PD induced by rotenone, *α*-lipoic acid showed similar results to those found in this study, and it was able to increase the number of crossings in the horizontal exploration when compared with the 6-OHDA-lesioned group without further treatment. This confirms the improvement in motor performance in the open field test produced by *α*-lipoic acid.

The cylinder test is used as a tool for evaluate fine motor activity. In this study, healthy animals used both paws simultaneously to touch the wall of the cylinder while animals pretreated with 6-OHDA touched the cylinder wall only with the paw ipsilateral to the lesion.

A similar result was reported in the study developed by Jouve et al. [36] which studied the anti-parkinsonian effect of deep brain stimulation in a PD model induced by 6-OHDA. In this study the control group showed a greater use of both paws simultaneously to touch the wall of the cylinder while the group pretreated with 6-OHDA showed a significant reduction in the use of both paws simultaneously.

This study showed that the *α*-lipoic acid was able to promote an improvement in fine motor function, since the animals pretreated with 6-OHDA that received *α*-lipoic acid touched more times the cylinder wall with the paw contralateral to the lesion as compared to the group pre-treated with 6-OHDA without further treatment.

The unilateral lesion caused by 6-OHDA promotes a chronic deficit in sensorimotor and somatosensory functions and in the use of the contralateral paw, generating an asymmetry in the use of the forelegs in the cylinder test, which involves greater use of the paw ipsilateral to the lesion. The maximum and exclusive use of the ipsilateral paw indicates a depletion of DA levels in the nigrostriatal pathway [13].

The origin of neurodegeneration that occurs the PD is still not well understood, however, it is known that some events are involved in the development of this pathology, among them is the oxidative stress. Evidence suggests that mitochondrial dysfunction, accumulation of Fe^2+^, lipid peroxidation and antioxidant system failure are part of this process [37, 38].

This study investigated the possible neuroprotective effect of *α*-lipoic acid in the model of PD induced by 6-OHDA. The *α*-lipoic acid is an antioxidant and acts to reduce the oxidation and lipid peroxidation.

The direct inhibition of 6-OHDA on the electron transport chain culminates with the decrease of ATP, leading to severe mitochondrial dysfunction. Such dysfunction promotes changes in mitochondrial membrane permeability resulting in loss of electrons and contributing to the formation of free radicals in dopaminergic neurons leading to a destruction of them in a progressive and irreversible way [39].

The nervous system is more sensitive to the damaging action of free radicals than other tissues in our body, once the metabolism of the brain is extremely high which favors the constant formation of reactive oxygen and nitrogen species. Besides that, the antioxidant defense system is not too efficient in the removal of these agents, favoring the neurodegeneration [40, 41].

In this study 6-OHDA promoted increased levels of MDA in the TBARS test indicating that there was an increase of lipid peroxidation in the striatum. Another interesting result is that oxidative damage was not restricted to this brain area, but also struck the prefrontal cortex and hippocampus. This demonstrates that the progressive destruction produced by 6-OHDA is not restricted only to neurons of the nigrostriatal pathway.

The results of this study are consistent with other research showing that the 6-OHDA has deleterious effects on many brain regions such as prefrontal cortex, cerebellum and hippocampus, the latter being the first one to suffer the effects of 6-OHDA in the induction of oxidative process, even before the onset of motor impairment resulting from loss of regulation by the nigrostriatal pathway [42, 43].

The *α*-lipoic acid showed a potential neuroprotective effect, once it was able to reduce the lipid peroxidation in TBARS test in the striatum and in the prefrontal cortex and hippocampus. Similar results were observed in a study by Zaitone et al. [35] using the model of rotenone where *α*-lipoic acid also reduced the levels of thiobarbituric acid reactive substances, acting as a protective agent.

Lipid peroxidation is the broadest process of oxidative damage, promoting the breakdown of cell membrane lipids and the formation of peroxyl radical. Once started the event, this one spreads inducing cell destruction chain [44, 45].

Still regarding lipid peroxidation, treatment with L-DOPA caused a significant increase of MDA levels when compared to the group pre-treated with 6-OHDA without further treatment. This finding suggests that L-DOPA has pro-oxidant effect, increasing the formation of free radicals, which culminates with the progression of neurodegeneration. The association of *α*-lipoic acid with L-DOPA reversed this effect of L-DOPA and again, it is evident the possible neuroprotective effect of this antioxidant.

 Di Stefano et al. [46] showed that the combined use of *α*-lipoic acid and L-dopa improved motor function in mice in the open field test, as well as promoted a significant reduction of oxidative stress once it reduced the formation of free radicals and improved the endogenous antioxidant defense system.

L-DOPA is the standard treatment to control the motor dysfunction in PD. However, chronic use of this drug can cause severe damage, such as the emergence of dyskinesias and a worsening of neurodegeneration of dopaminergic terminals. This mechanism is related to increase extracellular DA resulting from the metabolism of L-DOPA, as well as from the reduced activity of this catecholamine transporter (DAT). The accumulation of this neurotransmitter promotes hyperexcitation of D1 receptors as well as changes in expression of these receptors, being involved directly in motor exacerbating [47].

Studies show that L-DOPA and its metabolites promote oxidative stress [47, 48]. This mechanism is directly related to the fact that L-DOPA lead to decreased activity of complex I of mitochondrial chain in rats as demonstrated in the research by Przedborski et al. [49].

The L-DOPA is auto-oxidized promoting the formation of reactive oxygen species in the presence of Fe^2+^ by the Fenton reaction. Among the most produced are: hydrogen peroxide, hydroxyl radical, superoxide anion and others. Besides increasing the production of free radicals, L-DOPA acts to reduce the activity of the antioxidant enzymes catalase, glutathioneperoxidase (GPx), superoxide dismutase (SOD). This imbalance between production and elimination of radicals culminates with oxidative stress, favoring the progression of the disease [47, 50, 51].

It is well known that L-DOPA increases the production of dopamine (DA) in nigral dopaminergic neurons, while paradoxically inhibiting the firing of these neurons due to activation of D2 auto-receptors by extracellularly released DA. Using a combination of electrophysiology and calcium microfluorometry in brain slices, Guatteo et al. [52] demonstrated that L-DOPA has dual, inhibitory and excitatory, effects on nigral dopaminergic neurons, and suggested that the excitation and calcium rise may have long-lasting consequences for the activity and survival of these neurons when the expression or function of D2 receptors is impaired.

Studies have described excitotoxic effects of the L-DOPA in a variety of cell culture models. The excitotoxic effects were largely due not to L-DOPA itself, but to its nonenzymatic autoxidation product 2,4,5-trihydroxyphenylalanine (TOPA) quinone which is a potent activator of AMPA/kainate receptors [53–55].

It has been reported that both L-DOPA and DA can cause a prolonged rise of [Ca^2+^]_*i*_ in dopaminergic SNc neurons. The mechanism of the L-DOPA-induced long-lasting increase of [Ca^2+^]_*i*_ in dopaminergic SNc neurons still remains unclear. However, L-DOPA is converted to DA, and gradual degradation of DA by monoamine oxidase is directly associated with production of H_2_O_2_ which may lead to Ca^2 +^ influx through oxidative stress-sensing channels such as TRPM2 or TRPC5 [56] both expressed in the substantia nigra [57]. Consequently, dopaminergic neurons are in permanent state of oxidative stress, and this imbalance could lead to reduced levels of endogenous antioxidants [58, 59].

The *α*-lipoic acid is considered one of the most potent endogenous defense systems to combat the formation of reactive oxygen and nitrogen species, besides promoting the renewal of other antioxidants such as vitamin E, C, A, and coenzyme Q10 and reduced GSH, as well as act as a metal chelator [60–62].

Another important factor that classifies *α*-lipoic acid as a universal antioxidant is that it acts both in aqueous and greasy once it is amphipathic and may promote its antioxidant effect in the cytosol, in plasma membranes, in plasma and in lipoproteins [62, 63].

The DHLA is the reduced form of *α*-lipoic acid that interacts predominantly with the reactive oxygen species, but the oxidized form of *α*-lipoic acid may also inactivate free radicals. LA is found naturally in mitochondria on the E2 subunit acting as a cofactor for the enzyme pyruvate dehydrogenase and *α*-ketoglutarate dehydrogenase. Moreover, the beneficial action of LA may result from their ability to reduce the nicotinamide adenine dinucleotide phosphate oxidase (NADPH) and enhance the expression of antioxidant enzymes [64].

Our results showed that *α*-lipoic acid was able to promote a decrease in concentrations of nitrite and nitrate in the prefrontal cortex, hippocampus and striatum of 6-OHDA-lesioned rats.

Bilska et al [11] also demonstrated that the *α*-lipoic acid was able to reduce the production of nitric oxide and S-nitrosothiol in the prefrontal cortex and striatum of rats submitted to reserpine. Thus, the LA may have potential therapeutic value in PD.

Nitric oxide (NO) is a gas that is synthesized from L-arginine in a reaction catalyzed by the enzyme nitric oxide synthase (NOS). This gas plays an important role in physiological processes, however, in presence of superoxide anion, nitric oxide is converted into a potent free radical: peroxynitrite which is an agent extremely harmful to cells [11].

Another important free radical present in the PD model induced by 6-OHDA is H_2_O_2_. This neurotoxin undergoes auto-oxidation in the presence of molecular oxygen leading to the formation of this injurious agent [65].

H_2_O_2_ resulting from auto-oxidation of 6-OHDA in the presence of iron can be converted to the hydroxyl radical by the Fenton reaction, which is a free radical more harmful to cells. The mesencephalic dopaminergic neurons which are rich in iron due to its accumulation by neuromelanin favors the attraction/selectivity for 6-OHDA, being a potential target of this toxin [65].

The *α*-lipoic acid is capable of reducing the formation of H_2_O_2_ as shown in this research, once the groups treated with this antioxidant decreased the activity of catalase, an enzyme responsible for removing free radicals, compared to the group 6-OHDA-lesioned.

The free oxygen leads to formation of superoxide anion which is catalytically dismuted by the action of SOD in H_2_O_2_, this in turn is metabolized by catalase and GPx into water and oxygen. These enzymes have the main function to prevent the conversion of these radicals in the radical-OH, which is as harmful as peroxynitrite [66].

As an antioxidant *α*-lipoic acid works by removing the hydroxyl radicals, hydrogen peroxide in its free form, superoxide and peroxynitrite. Due to the potent effect of *α*-lipoic acid, it would also be capable of preventing neuronal damage caused by reactive species derived from the oxygen produced during neurodegenerative diseases. It was also described an important antioxidant action for this compound in reducing the inflammatory process in the brain induced by free radicals [67].

The anti-inflammatory action of *α*-lipoic acid and DHLA occurs by regulating the redox state in cells affecting gene expression and transcription of pro-inflammatory factors such as NFKB and AP1 oxidatively induced [64, 68].

Another important activity of *α*-lipoic acid and its reduced form, the DHLA is its activity as a chelator of metals such as iron, copper, manganese and zinc. These metals can cause oxidative damage by favoring formation of reactive oxygen and nitrogen species. Bush [69] found that DHLA promoted chelation of iron and copper in the brain, showing a neuroprotective effect on Alzheimer's disease by reducing the injuries caused by free radicals.

In fact, *α*-lipoic acid has shown to be able to reduce injuries associated with intracerebral injection of 6-OHDA. The decreased formation of thiobarbituric acid reactive substances, the decrease in catalase activity and reduced production of nitrite could explain the protective action of this drug.

## 5. Conclusion

The *α*-lipoic acid had a protective action against the lesion induced by 6-OHDA evaluated by behavioral tests and oxidative stress, suggesting its possible use as adjuvant in Parkinson's disease treatment.

## Figures and Tables

**Table 1:** Evaluation of the effects of *α*-lipoic acid alone or combined with L-DOPA on the cylinder test in rats subjected to pre-treatment with 6-OHDA.

Group	Paw contralateral to the lesion	Paw ipsilateral to the lesion	Double paws
Control	7.5%	10.2%	82.3%
6-OHDA	0.0%	100%^a^	0.0%^a^
6-OHDA + L-DOPA	4.59%	91.41%^a^	4%^a^
6-OHDA + LA100	16.2%^abc^	61.9%^abc^	21.9%^abc^
6-OHDA + LA200	18.3%^abc^	66.7%^abc^	15.0%^abc^
6-OHDA + LA100 + L-DOPA	31.3%^abcd^	15.9%^bcd^	52.8%^abcd^
6-OHDA + LA200 + L-DOPA	17.2%^abcf^	82.8%^af^	0.0%^aef^

Values are expressed as a percentage of the number of observations. Eight animals per group were used. ^a^versus control, ^b^versus 6-OHDA, ^c^versus 6-OHDA + LDOPA, ^d^versus 6-OHDA + LA100, ^e^versus 6-OHDA + LA200, ^f^versus 6-OHDA + LA100 + LDOPA com *P* < 0.0001 (One-way ANOVA and Tukey as the *post hoc* test).
